# Supplementary material for: Bile acid-gut microbiota imbalance in cholestasis and its long-term effect in mice
Source: mSystems. 2024 Jun 27;9(7):e00127-24. doi: 10.1128/msystems.00127-24 (PMC11265269; doi:10.1128/msystems.00127-24)
Supplement: Supplemental Figures — Figure S1-S6. [file msystems.00127-24-s0003.pdf]

Supplementary Figures

Figure S1

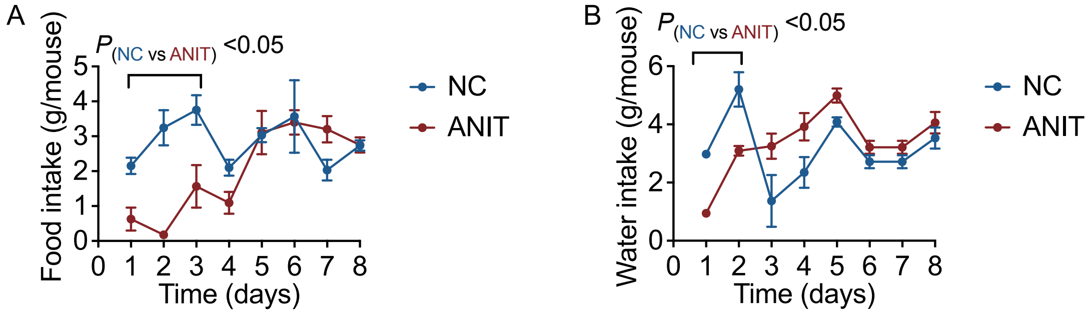

Fig. S1 | (A) Food intake, and **(B)** drinking water of NC and ANIT mice, n=10/group. NC, the control mice; ANIT, the mice with 75 mg/kg ANIT gavage. The data in **(A)-(B)** are shown as mean ± s.e.m., and Student's t-test (two-tailed) was used to analyze differences between groups. *P <0.05 and **P <0.01.

Figure S2

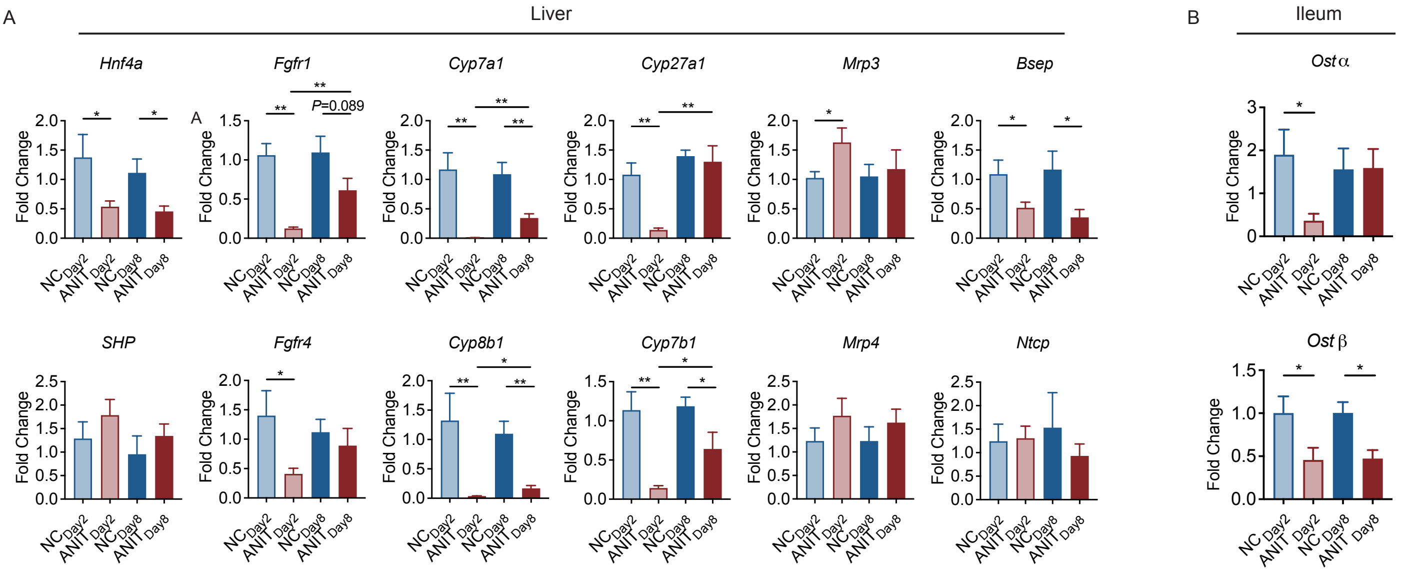

Fig. S2 | Levels of mRNA expression of genes related to transport of bile acids in the **(A)** liver and **(B)** ileum, n=5/group. NC, the control mice; ANIT, the mice with 75 mg/kg ANIT gavage. The data in **(A)-(B)** are shown as mean ± s.e.m., and Student's t-test (two-tailed) was used to analyze differences between groups. *P <0.05 and **P <0.01.

Figure S3

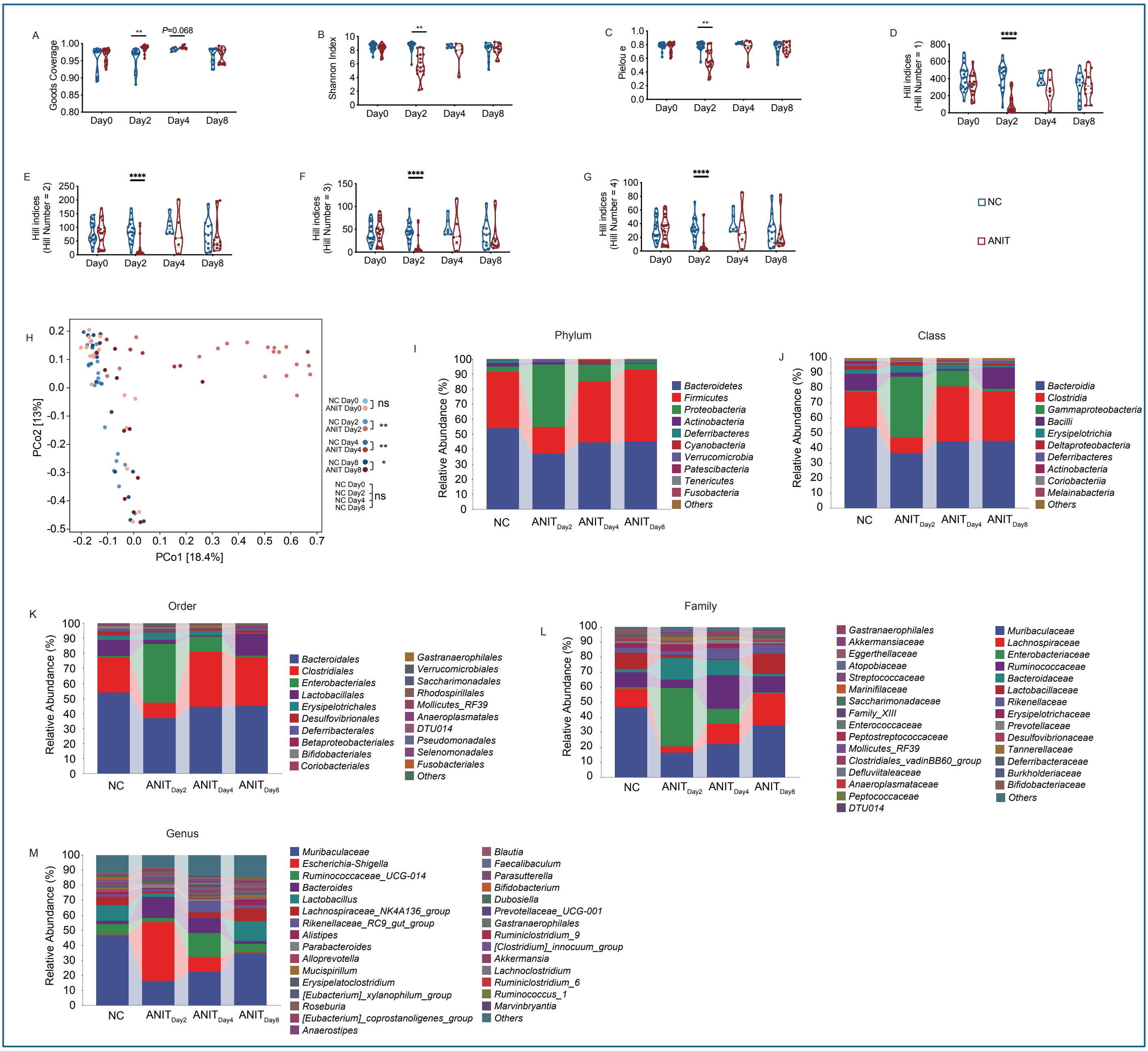

Fig. S3 | (A)-(G) The alpha-diversity of gut microbiota on day 2, 4 and 8 of the fecal samples from NC and ANIT groups. **(H)** Overall gut microbial structure in NC and ANIT mice [Unlike the figure 2C in the main text, the samples from the NC mice are separated at each time point]. Principal coordinate analysis (PCoA) was performed based on Bray-Curtis distance at the amplicon sequence variant (ASV) level. The distribution of gut bacteria at the **(I)** phylum, **(J)** class, **(K)** order, **(L)** family, and **(M)** genus levels. NC_{Day2}, day 2 of the control mice; NC_{Day4}, day 4 of the control mice; NC_{Day8}, day 8 of the control mice after the commence of the experiment; $ANIT_{Day2}$, day

2 of the mice after 75 mg/kg ANIT gavage; $ANIT_{Day4}$, day 4 of the mice after 75 mg/kg ANIT gavage; $ANIT_{Day8}$, day 8 of the mice after 75 mg/kg ANIT gavage. The data in **(A)-(G)** are expressed as mean ± s.e.m., and Student's t-test (two-tailed) was used to analyze differences between groups. *P <0.05, **P <0.01 and ****P <0.0001.

Figure S4

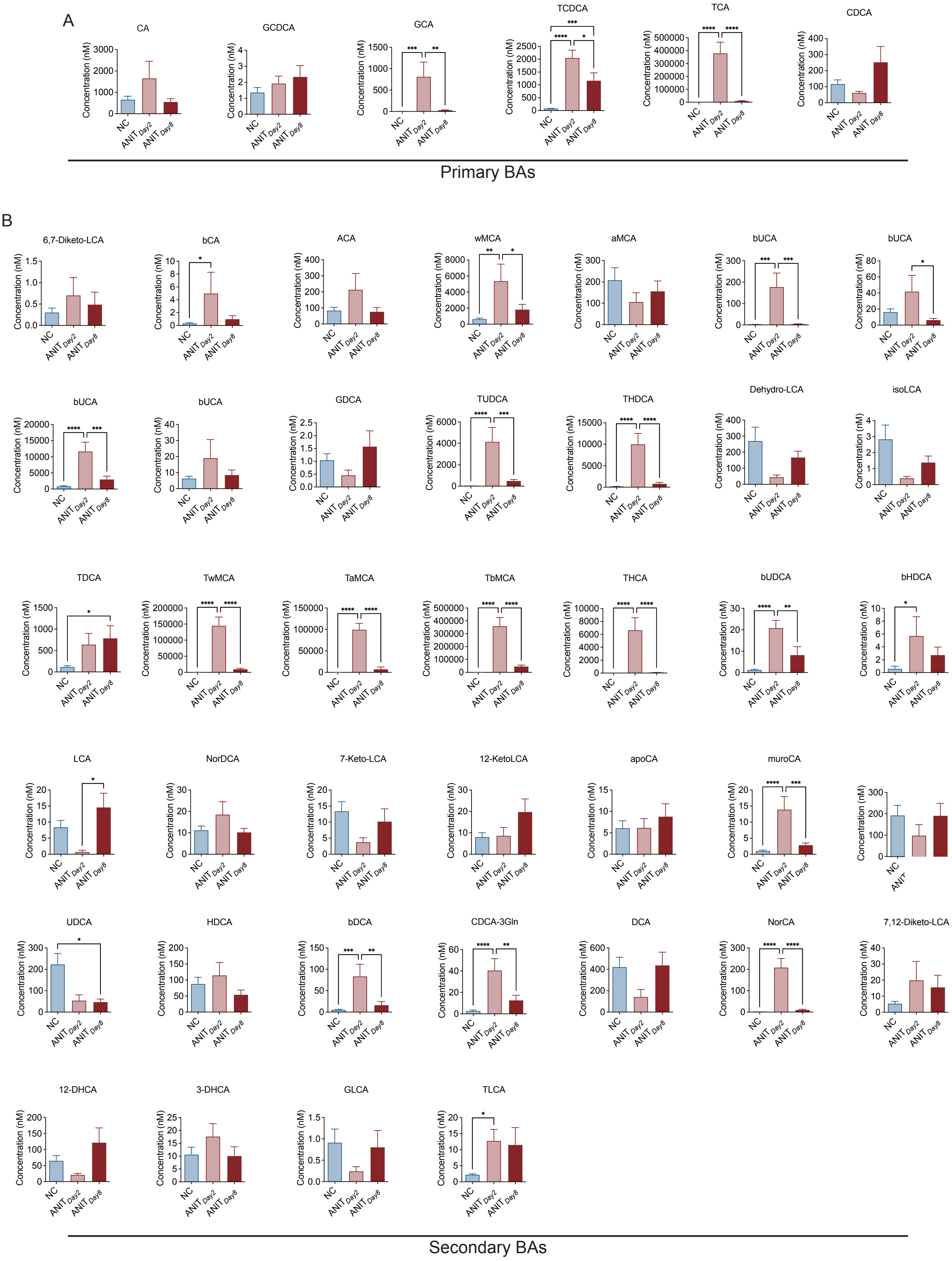
A
CA
GCDCA
GCA
TCDCA
TCA
CDCA
Concentration (nM)
NC
ANIT Day2
ANIT Day8
Primary BAs
B
6,7-Diketo-LCA
bCA
ACA
wMCA
aMCA
bUCA
bUCA
bUCA
bUCA
GDCA
TUDCA
THDCA
Dehydro-LCA
isoLCA
TDCA
TwMCA
TaMCA
TbMCA
THCA
bUDCA
bHDCA
LCA
NorDCA
7-Keto-LCA
12-KetoLCA
apoCA
muroCA
UDCA
HDCA
bDCA
CDCA-3Gln
DCA
NorCA
7,12-Diketo-LCA
12-DHCA
3-DHCA
GLCA
TLCA
Secondary BAs

Fig. S4 | Bile acid measurement. Serum **(A)** primary bile acid and **(B)** secondary bile acids concentration detected by UPLCMS/MS in each sample from control and ANIT treated mice, n=5/group. NC, the control mice; ANIT, the mice with 75 mg/kg ANIT gavage. The data are shown as mean ± s.e.m., and one-way ANOVA was used to analyze differences between groups. **P* <0.05 and ***P* <0.01, ****P*<0.001; *****P*<0.0001.

Figure S5

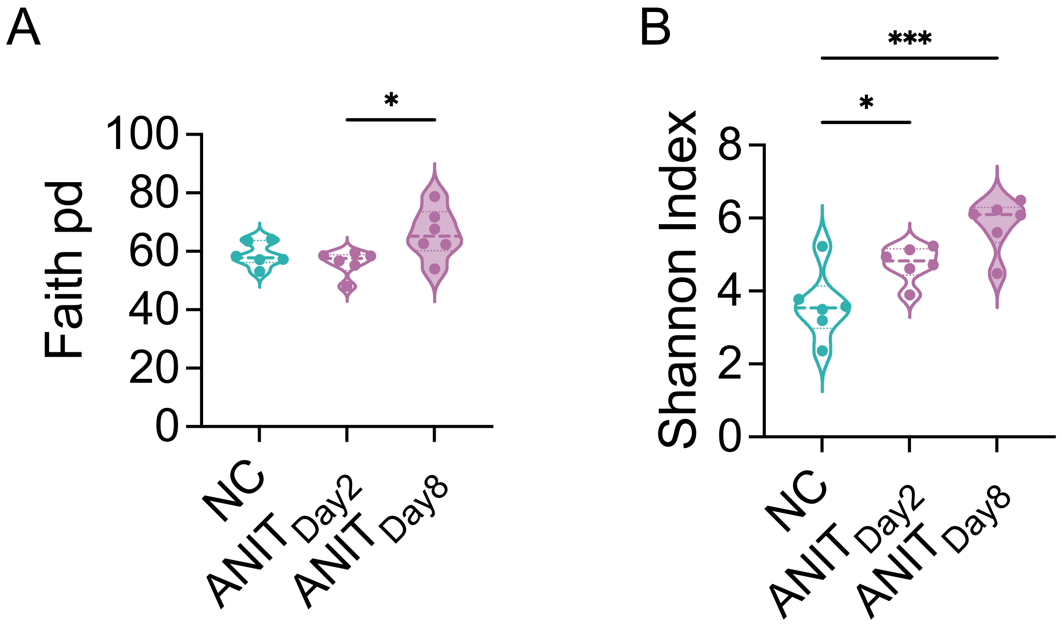

Fig. S5 | (A)-(B) The alpha-diversity of gut microbiota in the ileum content of NC and ANIT group on day 2 and 4; n=6/group. NC, the control mice; ANIT, the mice with 75 mg/kg ANIT gavage. The data are shown as mean ± s.e.m., and one-way ANOVA was used to analyze differences between groups. **P* <0.05 and ****P* <0.001.

Figure S6

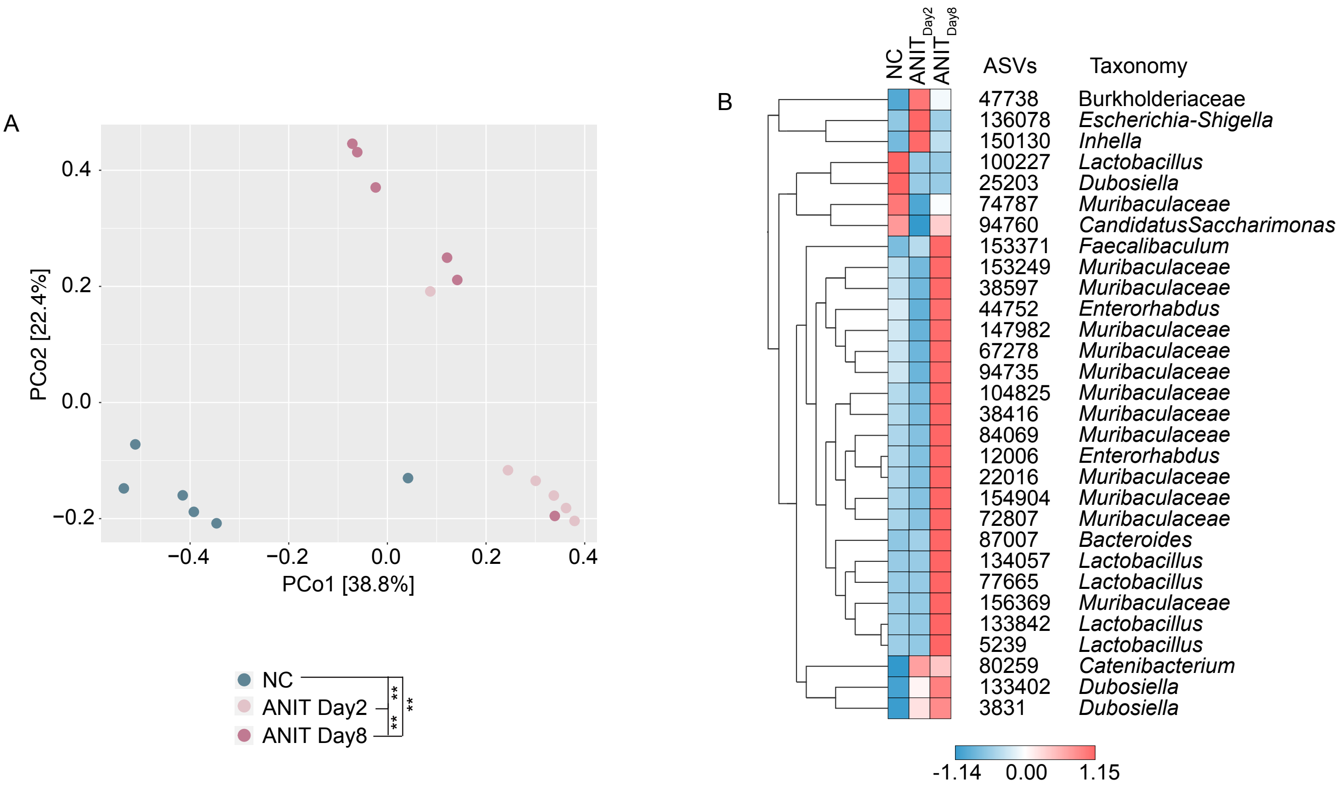

Fig. S6 | (A) Overall gut microbial structure in the ileum content of NC and ANIT mice. Principal coordinate analysis (PCoA) was performed based on Bray-Curtis distance at the amplicon sequence variant (ASV) level. **(B)** Thirty ASVs that were significantly altered after ANIT treatment, as identified using random forest models. The heat map shows the relative abundance (log_{10} transformed) of each ASV in samples from a group of mice. N=6/group;. NC, control group, $ANIT_{Day2}$, day 2 after 75 mg/kg ANIT gavage and $ANIT_{Day8}$, day 8 after 75 mg/kg ANIT gavage.
