# Supplementary material for: Bile acid-gut microbiota imbalance in cholestasis and its long-term effect in mice
Source: mSystems. 2024 Jun 27;9(7):e00127-24. doi: 10.1128/msystems.00127-24 (PMC11265269; doi:10.1128/msystems.00127-24)
Supplement: Supplemental Legends — Supplemental figure, table, and file legends. [file msystems.00127-24-s0004.docx]

**Supplementary Figure** **Legends**

**Fig. S1 | (A)** Food intake, and **(B)** drinking water of NC and ANIT mice, n=10/group. NC, the control mice; ANIT, the mice with 75 mg/kg ANIT gavage. The data in **(A)**-**(B)** are shown as mean ± s.e.m., and Student’s t-test (two-tailed) was used to analyze differences between groups. **P* <0.05 and ***P* <0.01.

**Fig. S2 |** Levels of mRNA expression of genes related to transport of bile acids in the **(A)** liver and **(B)** ileum, n=5/group. NC, the control mice; ANIT, the mice with 75 mg/kg ANIT gavage. The data in **(A)**-**(B)** are shown as mean ± s.e.m., and Student’s t-test (two-tailed) was used to analyze differences between groups. **P* <0.05 and ***P* <0.01.

**Fig. S3 | (A)-(G)** The alpha-diversity of gut microbiota on day 2, 4 and 8 of the fecal samples from NC and ANIT groups. **(H)** Overall gut microbial structure in NC and ANIT mice [Unlike the figure 2C in the main text, the samples from the NC mice are separated at each time point]. Principal coordinate analysis (PCoA) was performed based on Bray-Curtis distance at the amplicon sequence variant (ASV) level. The distribution of gut bacteria at the **(I)** phylum, **(J)** class, **(K)** order, **(L)** family, and **(M)** genus levels. NC_Day2_, day 2 of the control mice; NC_Day4_, day 4 of the control mice; NC_Day8_, day 8 of the control mice after the commence of the experiment; ANIT_Day2_, day 2 of the mice after 75 mg/kg ANIT gavage; ANIT_Day4_, day 4 of the mice after 75 mg/kg ANIT gavage; ANIT_Day8_, day 8 of the mice after 75 mg/kg ANIT gavage. The data in **(A)-(G)** are expressed as mean ± s.e.m., and Student’s t-test (two-tailed) was used to analyze differences between groups. **P* <0.05, ***P* <0.01 and *****P* <0.0001.

**Fig. S4 | Bile acid measurement.** Serum **(A)** primary bile acid and **(B)** secondary bile acids concentration detected by UPLCMS/MS in each sample from control and ANIT treated mice, n=5/group. NC, the control mice; ANIT, the mice with 75 mg/kg ANIT gavage. The data are shown as mean ± s.e.m., and one-way ANOVA was used to analyze differences between groups. **P* <0.05 and ***P* <0.01, ****P*<0.001; *****P*<0.0001.

**Fig. S5 | (A)-(B)** The alpha-diversity of gut microbiota in the ileum content of NC and ANIT group on day 2 and 4; n=6/group. NC, the control mice; ANIT, the mice with 75 mg/kg ANIT gavage. The data are shown as mean ± s.e.m., and one-way ANOVA was used to analyze differences between groups. **P* <0.05 and ****P* <0.001.

**Fig. S6 | (A)** Overall gut microbial structure in the ileum content of NC and ANIT mice. Principal coordinate analysis (PCoA) was performed based on Bray-Curtis distance at the amplicon sequence variant (ASV) level. **(B)** Thirty ASVs that were significantly altered after ANIT treatment, as identified using random forest models. The heat map shows the relative abundance (log_10_ transformed) of each ASV in samples from a group of mice. N=6/group;. NC, control group, ANIT_Day2_, day 2 after 75 mg/kg ANIT gavage and ANIT_Day8_, day 8 after 75 mg/kg ANIT gavage.

**Supplementary Table Legends**

**Table S1** | Primer list for qPCR.

**Table S2** | Abundance matrix of genes encoding virulence factors in fecal samples.

**Table S3** | Disease-related coding gene information annotated in the draft genome.

**Table S4** | Information on encoding virulence factors genes annotated in the draft genome.

**Table S5** | The CheckM calculation results for all bins.

**Table S6** | The results of the species-level annotation and classification of the bins.

**Other Supplementary File Legends**

**Supplementary file 1:** The 16S rRNA Sequence of ASV136078.

**Supplementary file 2:** The draft genome of ASV136078.
